# Alcohol beverage control, privatization and the geographic distribution of alcohol outlets

**DOI:** 10.1186/1471-2458-12-1015

**Published:** 2012-11-21

**Authors:** Tony H Grubesic, Alan T Murray, William Alex Pridemore, Loni Philip Tabb, Yin Liu, Ran Wei

**Affiliations:** 1 Geographic Information Systems and Spatial Analysis Laboratory, College of Information Science and Technology, Drexel University, Philadelphia, PA, 19104, USA; 2 GeoDa Center for Geospatial Analysis and Computation, School of Geographical Sciences and Urban Planning, Arizona State University, Tempe, AZ, 85287, USA; 3 Department of Criminal Justice, Indiana University, Bloomington, IN, 47405, USA; 4 Department of Epidemiology and Biostatistics, School of Public Health, Drexel University, Philadelphia, PA, 19102, USA

**Keywords:** Alcohol beverage control, Alcohol outlets, Alcohol availability, Location modeling, GIS

## Abstract

**Background:**

With Pennsylvania currently considering a move away from an Alcohol Beverage Control state to a privatized alcohol distribution system, this study uses a spatial analytical approach to examine potential impacts of privatization on the number and spatial distribution of alcohol outlets in the city of Philadelphia over a long time horizon.

**Methods:**

A suite of geospatial data were acquired for Philadelphia, including 1,964 alcohol outlet locations, 569,928 land parcels, and school, church, hospital, park and playground locations. These data were used as inputs for exploratory spatial analysis to estimate the expected number of outlets that would eventually operate in Philadelphia. Constraints included proximity restrictions (based on current ordinances regulating outlet distribution) of at least 200 feet between alcohol outlets and at least 300 feet between outlets and schools, churches, hospitals, parks and playgrounds.

**Results:**

Findings suggest that current state policies on alcohol outlet distributions in Philadelphia are loosely enforced, with many areas exhibiting extremely high spatial densities of outlets that violate existing proximity restrictions. The spatial model indicates that an additional 1,115 outlets could open in Philadelphia if privatization was to occur and current proximity ordinances were maintained.

**Conclusions:**

The study reveals that spatial analytical approaches can function as an excellent tool for contingency-based “what-if” analysis, providing an objective snapshot of potential policy outcomes prior to implementation. In this case, the likely outcome is a tremendous increase in alcohol outlets in Philadelphia, with concomitant negative health, crime and quality of life outcomes that accompany such an increase.

## Background

This paper details an exploratory spatial analytical approach for estimating the impacts of privatization on alcohol outlet distribution in an urban environment. The emergence and distribution of alcohol outlets is the byproduct of many factors, including public policy, market forces, and neighborhood collective efficacy. In turn, empirical research demonstrates that higher outlet density and alcohol availability are associated with a number of negative social and health outcomes, including higher rates of violence [1-5], youth drinking and driving [6,7], alcohol-related automobile crashes [8], child abuse and neglect [9], youth suicide [10], and sexually transmitted diseases [11]. Thus, developing strategies for evaluating the spatial distribution of outlets is important for quality of life, public safety, and public health.

One strategy for evaluating the distribution of alcohol outlets is to use spatial analytical methods that enable assessment of policies, land use restrictions, and spatial relationships. Examples include siting critical infrastructure [12], managing ecosystems for endangered species recovery [13], and managing convicted sex offenders [14]. Rather than retrospectively evaluating the impact of a policy once it is already in place, one useful way of implementing such approaches is to apply them prospectively, thereby providing planners and policymakers with a tool to estimate outcomes prior to implementation [14].

In this paper we apply spatial analytical techniques to evaluate the current and estimate the future distribution of alcohol outlets in Philadelphia, Pennsylvania. We chose this case study for specific reasons: the distribution of alcohol outlets has important ramifications for quality of life and public health, and Pennsylvania is considering a move away from an Alcohol Beverage Control state to a privatized alcohol distribution system [15]. Privatization, which essentially removes state control and its associated monopoly on alcohol sales, would have profound implications for alcohol availability, outlet distribution, and a variety of public health and safety outcomes. The goals of our analysis are to (1) examine the current alcohol outlet distribution in Philadelphia to provide a benchmark from which assessment of ordinances restricting alcohol outlet placement is possible and (2) estimate prospectively the impact of alcohol license privatization on the number and placement of alcohol outlets. The development of this framework and associated analysis is important for two main reasons. First, the ability to benchmark the existing distribution of alcohol outlets and compare it against existing policy guidelines is an important first step in understanding features, including biases, of the current licensing system. Second, if privatization were to occur, the existing distribution of outlets can be compared against alternative scenarios, where there are fewer controls on the number and location of outlets and retailers, reflecting a deregulated urban market. Further, this type of contingency analysis allows the public health implications of alcohol availability in a community to be evaluated prior to policy change.

### Alcohol beverage control

In 2010 there were 21 alcoholic beverage control (ABC) states in the U.S. State beverage control agencies possess wide-ranging powers. In addition to setting prices and determining which brands and products may be sold in regulated stores, ABC states can set restrictions on where, when, and how beverages are sold [16]. For example, the Pennsylvania Liquor Control Board (PLCB) is responsible for granting and issuing all licenses and permits for alcohol sales, and there are numerous regulations in the state. Among others, retail license holders are required to sell food; the property for which any retail license is sought cannot be owned by a manufacturer of liquor, malt, or brewed beverages unless the current owner had possession of the property prior to 1930; the premise may not be owned by the holder of a distributor or importing distributor license [17].

There are two basic models for alcoholic beverage controls at the state level. In license states (e.g., Ohio), licenses are issued for the sale of all types of alcoholic beverages (beer, wine, and spirits). In monopoly states (e.g., Pennsylvania), licenses are issued for the sale of beer and wine, but states retain control over the distribution of spirits (and sometimes wine) through officially sanctioned state stores. While there is debate over the impact of these two models on state economies, public health, and safety [16,18-20], empirical findings regarding outlet densities are resolute: alcohol outlet density is lower in ABC states [21].

### Privatization

In the context of alcohol beverage control, privatization refers to the elimination of state controlled monopolies in favor of commercial retailing. The implications of privatizing alcohol sales are significant. In Alberta, Canada, for example, within ten years of privatization the number of retail outlets tripled from 310 to 983 [22], substantially increasing access and dramatically escalating alcohol availability. This is a cause for concern for both law enforcement agencies and public health officials, and research on privatization in British Columbia reveals that alcohol-related deaths increased 3.25% for each 20% increase in private store density [23]. Given the negative consequences of privatizing alcohol sales, why are states motivated to follow this path? In Pennsylvania, there are two major reasons. First, recent estimates suggest an auction of wholesale and retail licenses by the PLCB would yield $1.1-$1.6 billion in immediate revenues [15]. Second, the current state administration headed by Governor Tom Corbett believes controlling and promoting the sales of alcohol are a conflict of interest [15].

Recent debates on the privatization of liquor sales in Virginia and Washington were fueled by a combination of interests. Aside from state governments’ interest in generating revenues, the private sector has an interest in privatization because it represents an opportunity for retailers to sell high volume, high profit items like beer and wine. Costco, a major retailer headquartered in Washington state, donated over $800,000 in cash and nearly $400,000 in in-kind contributions to promote privatization [24]. In June 2012, Washington was the first ABC state to privatize sales since Prohibition was repealed in 1933. In Virginia, during the 2009 gubernatorial race, Governor Bob McDonnell offered a plan to privatize the state’s system that would potentially yield nearly a half billion dollars to fund transportation projects. Under this plan, the number of stores selling spirits would triple, from 322 to 1,000, with licenses made available through auction to big box grocery, package, and convenience stores. Independent studies suggested the plan would not be feasible and would ultimately cost the state more money, including lost revenues, increased costs associated with law enforcement efforts (e.g., monitoring licenses), and higher costs for health care due to increased levels of alcohol consumption [24]. Today, Virginia remains an ABC state.

### Current licensing regulations in Pennsylvania

Under current regulations, outlets in Pennsylvania are managed by the PLCB, which grants several different license types, most falling under “retail.” Although space limitations prevent us from detailing all of the various types of licenses, generally speaking a retail license allows the sale of liquor, wine, and malt or brewed beverages for consumption on the license premises, while distributor licenses allow the sale of malt or brewed beverages by case lot (i.e., 24 12oz containers) [17].

All licenses are issued under Quota Laws, which establish the ratio of licenses that may be issued per number of county inhabitants. One retail license can be issued per 3,000 county residents. Hotels, airport restaurants, off-track wagering restaurants and certain golf course facilities *may* be issued a license in excess of the quota. One wholesale license can be issued per 30,000 residents in a Pennsylvania county. However, every municipality may vote to allow or prohibit the granting of retail liquor and/or dispenser licenses or wholesale licenses within their boundaries [17].

Given this regulatory backdrop, the remainder of the paper focuses on two major questions in a case study of Philadelphia. First, what is the distribution of retail alcohol licenses in Philadelphia? This is an important facet of the pre-privatization dialogue, providing context to potential changes in state laws and a key first step in understanding the characteristics and idiosyncrasies biases of the current licensing system. A related question at this stage is how this distribution of retail alcohol licenses compares to an alternative scenario where there are fewer controls on the number and location of outlets? Again, this type of contingency analysis allows the public health implications of alcohol availability to be evaluated prior to market privatization.

Regardless of the outcome concerning privatization in Pennsylvania, and beyond the benchmark provided by an examination of the distribution of alcohol outlets relative to the policies governing their placement, this study provides a tangible example of how spatial analytical techniques can be applied prospectively to an actual place (i.e., Philadelphia), in a state that is considering an actual policy change (i.e., privatization of retail alcohol licenses).

## Methods

The study area for our analysis was the city (and county) of Philadelphia, the fifth largest city in the U.S. with approximately 1.5 million people [25]. A range of exploratory methods were used in this study, including GIS, visualization and spatial optimization [26]. Parcel and land use data were obtained from the city of Philadelphia [27]. There were 569,928 parcels associated with the land use database for 2011.

Data on retail and wholesale liquor outlets for 2010 were obtained from the PLCB. Subsequent inquiries suggest that the number of licenses in Philadelphia (~2,000) is relatively stable. There are four major on-premise outlet types in Philadelphia: clubs (5.9%), distributors (5.6%), eating places (6.5%), and restaurants and bars (75%). All hotels, restaurants, and eating places are permitted to sell six-packs of beer for carry out and consumption off-premises, while distributors may only sell cases of beer (24 count) or single containers of at least 128 ounces for carry out. Airports, distilleries, large-scale distributors, and venues serving sacramental wine were excluded from our analysis, leaving 1,964 outlets identified by their corresponding land parcel.

Data on churches and hospitals were obtained from a national database of 12.5 million businesses [28]. Churches included all facility locations with a North American Industry Classification System (NAICS) code of 81311008 and 81311009. Hospitals corresponded to facilities with a NAICS code of 62211002. Due to difficulties establishing which businesses qualify as charitable institutions, these facilities were omitted from the analysis. Schools, public parks, and playgrounds were obtained from the city of Philadelphia [29], with parks and playgrounds supplemented with manual additions using the Google Earth database.

### Understanding the Pennsylvania liquor licensing system

In many ways, the PLCB and current state law treat alcohol outlets as undesirable facilities [30,31]. For the city of Philadelphia and elsewhere in Pennsylvania, the PLCB mandates that the premises for new or transferred licenses may not be within 200 feet of an existing licensed site and must be located at least 300 feet from schools, churches, hospitals, and playgrounds. The overarching goal of these dispersion laws is to ensure that sensitive institutions, neighborhoods and their associated populations (e.g., children and residents) do not suffer the negative outcomes associated with high-intensity alcohol retailing or consumption.

In the context of privatization, these types of guidelines represent a challenge. Retailers seek to maximize their profits, provided there is a sufficient market/demand. The surest way to maximize profits is to make outlets (and alcohol) widely accessible and available, a basic tenet of retail theory [32,33]. For example, the geographic market strategy for the global coffee chain Starbucks is to completely blanket a market area, even if stores cannibalize one another’s business [34,35]. Not only does this type of strategy maximize access and reduce delivery and management costs, it increases foot traffic for all stores in an area [34]. To a certain extent, dispersion ordinances limit access (and profits), effectively regularizing and thinning retail distributions, but it is widely recognized that some type of control is needed because the public health and quality of life implications of high intensity alcohol retailing and consumption are significant [2-6].

In this context, policy assessment requires retailer behavior to be translated or interpreted in the distribution of alcohol outlets. It is clear then that as many outlets as the market will bear can be expected to emerge under privatization, subject to established ordinances and land use restrictions. Further, it is possible to estimate the likely distribution of alcohol outlets over the long term using a spatial analytical model that mimics retailer behavior while imposing restrictions. The following notation is used in this modeling framework:

*k* = index of potential commercial parcels that can accommodate an alcohol outlet

α_*k*_= benefit of allocating a license to parcel *k*

*Γ*= minimum separation distance between alcohol outlets

*ϕ*_*k*_= commercial parcels within stipulated restriction distance Γ of parcel *k *(*j*|*d*_*kj*_ ≤ *Γ*)

*Z* = {_0_^1^ if a commercial parcel is allocated an alcohol outlet license otherwise

The formal model is as follows:

Maximize

(1) $$\sum_{k}\alpha_{k}Z_{k}$$

Subject to

(2) $$Z_{k}+Z_{j}\leq 1\;\forall k,j\in \varphi_{k}$$

(3) $$Z_{k}=\left\{0,1\right\}\;\forall k$$

The objective, equation (1), corresponds to retailer behavior that seeks to maximize outlet access, allocating licenses to as many commercial parcels as possible. The constraints in equation (2) prevent allocating a license to any two parcels that are too close to each other (i.e., within the minimum separation distance established by state ordinance). The constraints in equation (3) impose integer restriction decision variables: either a license is allocated to a parcel or it is not.

This model allows us to examine the expected distribution of alcohol outlets in the long term under a privatized market. The analysis can be thought of as a simulated licensing scenario, where there are fewer controls on the number and location of alcohol retailers and/or outlets. The model does not explicitly assume that existing outlets are “grandfathered” into the privatized system. Although this may be a reality in the short term, where existing alcohol outlets retain their licenses, the licensing process is a dynamic one. Outlets (including restaurants, distributors and eating places) are constantly opening and closing. Over time, many of the current outlets will no longer exist, being replaced by new licenses and locations that adhere to community and state guidelines and associated constraints. Second, for the purposes of this contingency analysis, licenses are only allocated to parcels that are designated for commercial activity. This means that licenses can be provided to existing big-box retailers (e.g., Sam’s Club), supermarkets, chain-pharmacies (e.g., CVS, Walgreens), restaurants and convenience stores (e.g., 7–11, Wawa) that occupy commercial space. Licenses can also be allocated to newly established retailers on commercial parcels only. It is also important to note that this modeling approach ensures that existing community standards are maintained, where outlets cannot be located within 200 feet of another licensed site and must be located at least 300 feet from schools, churches, hospitals, and playgrounds. The implications of these assumptions will be discussed later, as the existing distribution of alcohol outlets in Philadelphia is fraught with exceptions of all sorts.

## Results

**Figure 1:**
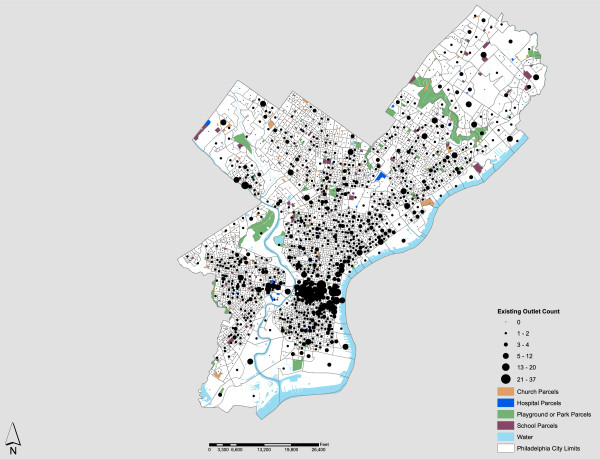
The Spatial Distribution of Alcohol Outlets in Philadelphia, by Census Block, 2010.

Figure 1 highlights the aggregate spatial distribution by census block of retail alcohol outlets (*n* = 1,964), schools (*n* = 376), parks and playgrounds (*n* = 122), hospitals (*n* = 39), and churches (*n* = 1,798) in Philadelphia. Clearly, the PLCB regulation stating that only one permit may be issued for every 3,000 residents in a county is currently not a primary consideration in Philadelphia. Under this guideline, only 508 licenses would be granted based on residential population.

There are several general descriptive statistics associated with the underlying distributions represented in Figure 1 worth noting. First, the average spatial density for the city, by block, is 28.44 per square mile and 0.105 per 1,000 feet of roadway. While neither statistic is particularly remarkable, there are several blocks that eclipse 800 outlets per square mile. Second, the average nearest neighbor distance (Euclidean) for alcohol outlets in Philadelphia is 404 feet. For comparative purposes, the average nearest neighbor distance for churches is 443 feet and 1,370 feet for school parcels. There are 507 church parcels in the city within 300 feet of an alcohol outlet. In part, these results address our first major question of identifying idiosyncrasies or interesting features associated with the existing distribution of alcohol outlets in Philadelphia. This suggests the PLCB regularly grants exceptions to prescribed quotas and separation requirements between outlets and community facilities. Unfortunately there are no public records or published guidelines to these exceptions or detailed evidence as to why they occurred. Another pattern of note is the high density of alcohol outlets in Center City Philadelphia, the largest entertainment and dining district in the city. Approximately 12% of all Philadelphia outlets are located in this area, though it is less than one square-mile in size and represents only six-tenths of one percent of the total land area of Philadelphia.

**Figure 2:**
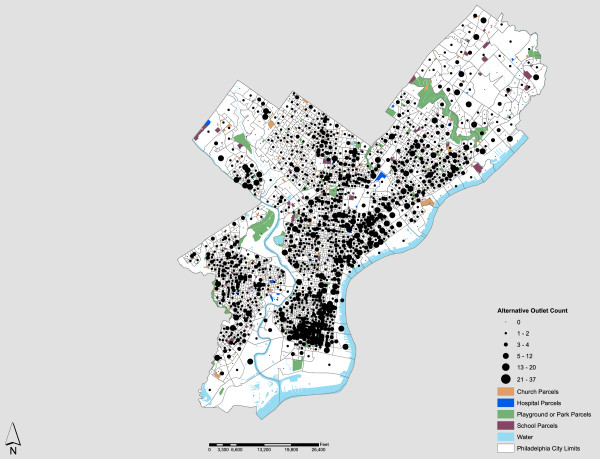
Modeled Future Distribution of Alcohol Outlets in Philadelphia Under Privatization.

Figure 2 shows an expected distribution pattern by census block that would emerge under the privatized license system in Philadelphia. This is obtained using the optimization model detailed previously. Figure 2 shows the number and location of alcohol outlets that would be possible if (1) alcohol license privatization occurred, (2) alcohol vendors located new outlets to maximize presence and availability, and (3) existing dispersion rules between outlets and sensitive community facilities were maintained. It is important to note that these changes would not occur immediately. Instead, the pattern represents a somewhat longer time horizon (e.g., a decade), where community guidelines and/or restrictions on outlets are fully realized for Philadelphia. One of the most significant differences between the observed and potential outlet distributions is the total number of outlets possible based on the license allocation criteria. There were 1,964 outlets in Philadelphia in 2010. Under privatization, the model suggests that some 3,079 outlets, representing a nearly 60% increase, would likely materialize. This is a dramatic difference, and the magnitude in geographic coverage associated with this increase is evident in Figure 2. There would be a significant infill of outlets in South Philadelphia, portions of West Philadelphia, and many areas north of Center City. Where density is concerned, the future distribution would be much higher, with nearly 46 outlets per square mile and 0.174 per 1,000 feet of roadway. This is a sharp increase from the existing distribution densities in Philadelphia, 28.44 and 0.105, respectively.

The major difference between the existing and potential distribution is that none of the outlet locations in the future would impinge upon the 300-foot restriction distance to community facilities or the 200-foot restriction distance between outlets set by the PLCB. Further, the overall reduction of outlet densities realized in Center City neighborhoods like Chinatown, Old City and Washington Square would likely help mitigate existing clusters of violence already present in these high density alcohol retailing areas [36]. In addition to representing expansion possibilities for PLCB licensed outlets under the current regulatory framework, community standards (i.e., distance restrictions) are more closely maintained because there are no exceptions to spatial proximity stipulations.

**Figure 3:**
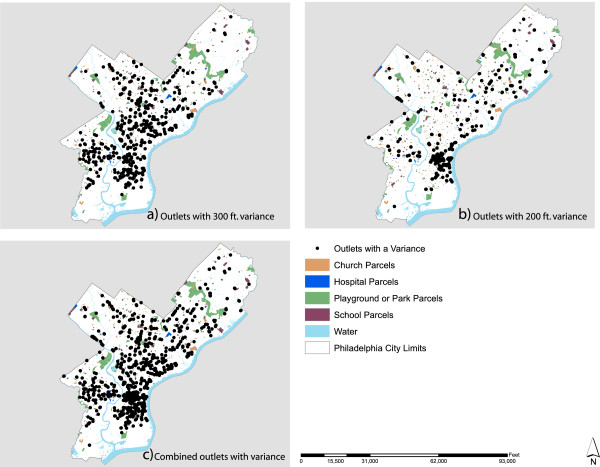
Spatial Distribution of Alcohol Outlets with a Variance in Philadelphia, 2010.

One last interesting facet of this analysis is a comparative evaluation of the existing distribution of outlets against the stated PLCB guidelines. There are currently 744 instances where alcohol outlets are within 300 feet of a parcel associated with a community facility (Figure 3a) and 854 alcohol outlets are within 200 feet of another outlet (Figure 3b). These totals do not represent unique sets: some outlets that are within 200 feet of each other are also within the 300 foot separation distance from community facilities and vice versa. When these overlaps are accounted for and all of the outlets are considered, 944 of the 1,964 outlets (48%) in Philadelphia require a variance by the PLCB (Figure 3c). That is, in nearly 50% of all cases the PLCB decided that outlets could violate the community standards pertaining to the spatial distribution of alcohol outlets. In other words, only about half of Philadelphia outlets adhere to PLCB community standards.

Finally, it is important to note that when one considers the spatial patterns displayed in Figures 2 and 3, where modeled outlets are regularized but many existing outlets require a variance for the dispersion ordinance, it is likely that the modeled tally of 3,079 outlets is conservative, at least for the short term. Again, although new outlets will emerge quickly, existing outlets can be persistent, as will their grandfathered variances to PLCB dispersion controls.

## Discussion

The negative social and health consequences associated with alcohol outlets are an important consideration for law enforcement, public health, and policymakers in the United States, making outlets, alcohol availability, and their impacts on the community important research topics. This paper provides a new perspective, leveraging the power of spatial analysis to describe the existing distribution of alcohol outlets and to estimate a scenario where there are fewer controls on the number and location of outlets in a major city grappling with privatization. Results reveal the existing distribution of licenses exhibits a number of idiosyncrasies, largely disregarding population-based quotas and often ignoring geographic regulations mandated by the Pennsylvania Liquor Control Board. Results also suggest Philadelphia could accommodate a much higher number of licenses if privatization occurs, even if current distance restrictions remained and were enforced. Further, it is clear that of all the PLCB constraints, the 1 outlet per 3,000 people rule is the least realistic under privatization. It is counterintuitive and makes no economic sense for the alcohol retailers. One of the major reasons Pennsylvania is considering privatization is to generate more tax revenues from alcohol sales. There is no reason for the state (or retailers) to adopt this rule, nor would it reflect the retail goals of market and sales maximization under privatization. In fact, recent evidence in the state of Washington, which privatized in June 2012 under Initiative 1183 (I-1183) and is encouraging a more laissez-faire retail system, suggests that while the price increase of 17% on alcohol sent some consumers across the state border to Oregon for their purchases [37], sales in Washington are way up – increasing 21% in July 2012 when compared to 2011 [38].^a^ Further, I-1183 allows for the state to gradually increase the number of spirits retailers to 1,428, relative to 328 outlets under the regulated system [39].

Given the recent developments in Washington and the continuing debate in Pennsylvania regarding privatization, our analysis also presents a number of pathways for exploring the implications of privatization in Philadelphia and beyond. First, the basic exploratory analyses suggest significant bias in the existing system with respect to PLCB guidelines limiting the proximity of outlets to community facilities like schools, hospitals, playgrounds, and churches. Variances to policy likely reflect decades of dynamic urban evolution and the changing composition of commercial environments throughout the city. For example, if an alcohol outlet was established in 1995 outside the 300-foot restriction distance to its nearest church but a new church locates within 300-feet of the existing outlet, this would be identified as regulatory variance. While outlet locations are generally more dynamic than these community facilities, Philadelphia has a long history and the persistence of historic outlets is an important consideration. For example, McGillin's Olde Ale House, located in Center City, has been operating since 1860, the year Abraham Lincoln was elected as president.

A second facet of our analysis pertains to the alternative license allocation scenario identified using the spatial model. The analysis suggested that 3,079 licenses/outlets would be permissible for Philadelphia under current guidelines. This 56% increase is likely to be greeted with disdain by law enforcement, public health officials, and many neighborhoods and citizens given existing evidence regarding the negative consequences associated with greater alcohol outlet density. This estimate is actually a conservative one, as it assumes current distance restrictions between outlets and sensitive community facilities (and between outlets and other outlets) were maintained and strictly enforced, and we have just shown this not to be the case (at least in Philadelphia). While it is impossible to predict how many licenses would be issued in Philadelphia if Pennsylvania was to privatize, or if there would be a formal limit on the outlet count, there is the potential for the number of outlets to grow more than the 56% suggested by the modeled case. One only needs to look to Alberta, where privatization led to a three-fold increase in alcohol outlets in a single decade [22]. The state of Washington is on the same path [39]. The reverse may also be true, Philadelphia could already be so saturated with alcohol outlets that no growth would occur and the number of outlets would continue to hover around 2,000. Regardless of the actual outcome, the value of this type of modeling approach is clear. Specifically, because the location model outlined in this paper is flexible enough to accommodate a wide variety of policy restrictions and parameters, it allows policymakers, public health officials, and law enforcement agencies to objectively evaluate *potential* outcomes *prior* to the implementation of new alcohol regulations, or in this case, a privatized state system. Thus, the type of insight it may offer is valuable in formulating policy, and over time the model could be fine-tuned to reflect likely outcomes based on previous experiences with regulation in comparable communities.

## Conclusions

Given the context we present in this paper, there are two reasonable alternatives for regulating alcohol licenses under privatization. First, Pennsylvania could dismiss all regulations, allowing local and regional markets to sort things out over time. This is the purest form of privatization, where the dynamics of outlet locations, product mix, pricing, and marketing efforts interact to make an outlet successful or not. Given the number and type of licenses that could be issued, a license to sell alcohol (from corner grocers to big-box retailers) would be a contributing factor in profitability. The second approach involves Pennsylvania maintaining existing (or some variation of) regulatory criteria for geographically managing the spatial distribution of alcohol outlets. Retaining regulations that allow officials to deny licenses in areas already dense with outlets is an important policy tool. Further, some order could be obtained when the state is inundated with requests for licenses after privatization. As noted by Pridemore and Grubesic [4], the ability to deny renewal applications for outlets with a history of regulatory or neighborhood-related problems is critical for maintaining community health and legal standards.

From a methodological perspective, it is important to reiterate that one appealing aspect of the spatial method detailed in this paper is its flexibility in accommodating different parameters for evaluating licensing scenarios for a major urban area. If privatization in Pennsylvania yielded a different set of geographic regulations, where distances varied by outlet types (e.g., off-premise outlets relative to bars) and the variety of sensitive facilities changed (e.g., other types of facilities were added to the list of protected sites), the model easily accommodates these modifications. This is important because rather than evaluating the impacts of licensing policies post hoc, one could apply this type of modeling as a prospective, contingency-based analysis. This type of “what-if” modeling provides policy-makers with an objective tool for determining potential policy outcomes prior to implementation [14]. Finally, this model scales nicely. While we focused on a relatively localized case study of Philadelphia, it is possible to apply this model to a much larger area, such as the entire Philadelphia metropolitan area or all of Eastern Pennsylvania. This is an important consideration when evaluating policies and associated outcomes over large and diverse geographic areas.

## Endnotes

^a^It is illegal for Pennsylvanians to purchase alcohol in border states (e.g., Delaware, New Jersey, etc.) and transport it back to the Commonwealth for consumption or sale. Specifically, Section 491(2) of the Liquor Code prohibits any person other than the Board, a manufacturer, or the holder of a sacramental wine license or of an importer license from possessing or transporting any liquor or wine within the Commonwealth of Pennsylvania which has not been purchased from a Pennsylvania wine and spirits store or a licensed limited winery [40]. Although there are minor exceptions for international purchases and some illicit market leakage to border states, legal controls in Pennsylvania make cross-border purchases risky for PA residents.

## Competing interests

The authors declare that they have no competing interests.

## Authors’ contributions

THG and ATM designed the study and participated in drafting the manuscript with WAP and LPT. RW and YL helped organize the data and coded several of the models in Gurobi and ArcGIS. All authors have approved the final manuscript.

## Pre-publication history

The pre-publication history for this paper can be accessed here:

http://www.biomedcentral.com/1471-2458/12/1015/prepub
